# Hyperprolactinaemia is common in Chinese premenopausal women with breast diseases

**DOI:** 10.3389/fgene.2023.1018668

**Published:** 2023-02-10

**Authors:** Jiang Zhu, Yuyi Tang, Cuixia Lv, Han Cong, Jie Liu, Song Zhao, Yawen Wang, Kai Zhang, Wenbin Yu, Qian Cai, Rong Ma, Jianli Wang

**Affiliations:** ^1^ Department of Breast Surgery, General Surgery, Qilu Hospital of Shandong University, Jinan, Shandong, China; ^2^ Department of General practice, Guangdong Second Provincial General Hospital, Guangzhou, Guangdong, China; ^3^ Shandong Center of Disease Control and Prevention, Jinan, Shandong, China; ^4^ Department of General Surgery, Qilu Hospital of Shandong University, Jinan, Shandong, China; ^5^ Department of Geriatric Medicine, Qilu Hospital of Shandong University, Jinan, Shandong, China; ^6^ Key Laboratory of Cardiovascular Proteomics of Shandong Province, Qilu Hospital of Shandong University, Jinan, Shandong, China; ^7^ Department of Gynaecology and Obstetrics, Qilu Hospital of Shandong University, Jinan, Shandong, China

**Keywords:** hyperprolactinaemia, breast lesions, premenopausal, Chinese women, prolactin (PRL)

## Abstract

**Purpose:** Hyperprolactinaemia has been proposed to play a role in breast lesions pathophysiology. Thus far, controversial results have been reported for the relationship between hyperprolactinaemia and breast lesions. Moreover, the prevalence of hyperprolactinaemia in a population with breast lesions is scarcely reported. We aimed to investigate the prevalence of hyperprolactinaemia in Chinese premenopausal women with breast diseases, and explore the associations between hyperprolactinaemia with different clinical characteristics.

**Methods:** This was a retrospective cross-sectional study performed in the department of breast surgery of Qilu hospital of Shandong University. Overall, 1,461 female patients who underwent the serum prolactin (PRL) level assay before breast surgery from January 2019 to December 2020 were included. Patients were divided into two groups: before and after menopause. Data were analyzed using SPSS 18.0 software.

**Results:** The results showed an elevated PRL level in 376 of the 1,461 female patients with breast lesions (25.74%). Furthermore, the proportion of hyperprolactinemia among premenopausal patients with breast disease (35.75%, 340/951) was significantly higher than among postmenopausal patients with breast disease (7.06%, 36/510). In premenopausal patients, the proportion of patients with hyperprolactinaemia and the mean serum PRL level were significantly higher in those diagnosed with fibroepithelial tumours (FETs) and in younger patients (aged < 35 years) than in those with non-neoplastic lesions and in those aged ≥ 35 years (both *p* < 0.05). Especially, the prolactin level exhibited steady ascending tendency for positive correlation with FET.

**Conclusion:** Hyperprolactinaemia is prevalent in Chinese premenopausal patients with breast diseases, especially in those with FETs, which implies a potential association, to some extent, between the PRL levels in various breast diseases.

## Introduction

Prolactin (PRL) is a protein hormone synthesised by lactotrophic cells in the adenohypophysis. PRL plays an important role in many biological processes including osmoregulation, immunomodulation, behaviour, reproduction, growth, and development (Freeman et al., 2000). PRL levels physiologically increase during pregnancy and lactation and in response to nipple stimulation, breast examinations, orgasm, or stress (Huynh et al., 2013; Savino, 2017; Alex et al., 2020). Pathological causes of hyperprolactinaemia include adenomas of the pituitary, benign or malignant tumours of the hypothalamus, infiltrative diseases of the hypothalamus (e.g., sarcoidosis), and injury of the hypothalamic pituitary stalk (Yuan and Wade, 1991; Anthony et al., 2016; Guo et al., 2019; Lv et al., 2019). Drug-induced hyperprolactinaemia commonly occurs in psychosis patients who take dopamine D2 receptor antagonists (Sykes et al., 2017). Gastrointestinal stimulants such as metoclopramide and domperidone can also elevate serum PRL levels through the mechanism of blocking dopamine receptor (Tonini et al., 2004). In addition, idiopathic hyperprolactinaemia (Hattori et al., 1992), macroprolactinaemia (Kalsi et al., 2019), chronic renal failure (Hou et al., 1985), hypothyroidism (Sharma et al., 2016), and loss-of-function mutation in the PRL receptor (PRLR) gene (Newey et al., 2013) are the pathophysiological conditions leading to the elevation of serum PRL levels in women.

The relationship between hyperprolactinaemia and breast lesions remains controversial. Although PRL could induce estrogen receptor-positive breast cancer (BC) in a murine model (Campbell et al., 2019), and initiate the transformation of normal ductal epithelial cells (Grible et al., 2021), many epidemiological studies have not shown an increased risk of BC in the population with hyperprolactinaemia (Dekkers et al., 2010; Berinder et al., 2011; Dekkers et al., 2015). Fibroepithelial lesions, also called fibroepithelial tumours (FETs), include fibroadenomas and phyllodes tumours, and they belong to a group of biphasic tumours with epithelial and stromal components (Van Osdol et al., 2014). PRL did not stimulate colony formation in primary cultured fibroadenoma (Manni et al., 1986). As shown in F344 rats, age-related hyperprolactinaemia might be responsible for the high background of fibroadenomas (Maier et al., 2012). Moreover, elevated serum PRL levels could be observed in patients with fibroadenomas or phyllodes tumours (Nicol et al., 2002).

In addition to palpable breast masses and mastalgia, nipple discharge is the most commonly encountered symptom in outpatient practice (Ma et al., 2016). Physiological discharge is often caused by galactorrhea associated with hyperprolactinaemia, while intraductal papillary neoplasms (comprising intraductal papilloma (IDP), atypical hyperplasia, and ductal carcinoma *in situ*) are the main causes for the pathological discharge (Huang and Molitch, 2012; Salzman et al., 2019). In one study, the neu-related lipocalin-PRL transgenic female virgin mice showed mammary developmental abnormalities, mammary intraepithelial neoplasias, and papillary adenocarcinomas (Rose-Hellekant et al., 2003). Ductal ectasia and granulomatous lobular mastitis are non-puerperal aseptic mastitis. Except for nipple retraction, ductal ectasia occurs partly because of increased PRL secretion (Peters and Schuth, 1989). Elevated levels of PRL after surgical treatment is an independent risk factor for the recurrence of granulomatous lobular mastitis (Huang and Wu, 2021).

The prevalence of hyperprolactinaemia has been widely investigated in certain populations such as hypothyroidism patients (Sirohi and Singh, 2018), antipsychotic drugs users (Wang et al., 2014), infertility patients (Souter et al., 2010; Ambulkar et al., 2021), and healthy premenopausal female donors (Alpanes et al., 2013). However, clinical data on hyperprolactinaemia prevalence in the population with breast lesions are scarcely reported in literature. In this study, we aimed to determine the prevalence of hyperprolactinaemia and unravel the associations between hyperprolactinaemia with different clinical characteristics in Chinese premenopausal women with breast diseases.

## Materials and methods

### Data source, patient collection, and ethics statement

In this study, we continuously assessed a large series of inpatients serum PRL levels in the breast surgery department of Qilu Hospital before operation from January 2019 to December 2020. In total, 1,467 patients had undergone the serum PRL level test and got pathological diagnosis. After excluding 6 male patients, 1,461 female patients were finally enrolled for further analysis. Patients were divided into two groups: before and after menopause. The postmenopausal criteria are defined as a time interval of more than one year from the time point of data collection to the last menstruation. Research was focus on exploring the associations between hyperprolactinaemia with different clinical characteristics in 951 premenopausal patients. All the patients’ clinical data were collected from the inpatient medical records system in Qilu Hospital of Shandong University. The study protocol was approved by the Ethical Committee of Qilu Hospital of Shandong University, and informed consent was obtained from the patients.

### Assays and criteria for the definition of hyperprolactinaemia

Fasting blood samples were drawn from each patient in the morning after admission. Serum PRL levels were tested in Roche cobas e601 using Elecsys Prolactin Ⅱ Kit (Ref. No. 03203093 190, Roche, CH) according to the manufacturer’s instruction. We considered a normal serum PRL concentration range from 4.79 ng/mL (102 μIU/mL) to 23.30 ng/mL (496 μIU/mL) in women, as recommended by the manufacturer of the PRL assay. Patients whose serum PRL levels higher than 23.30 ng/mL (Upper limit of reference value based on 300 healthy donors according to manufacturer’s instruction) were diagnosed with hyperprolactinaemia.

### Statistical analysis

Data were analysed using SPSS 18.0 software (IBM, United States). The shapiro-Wilk method was used for normality test, the data that did not conform to the normal distribution was logarithmic transformed to achieve approximate normality. Levene method was used to test homogeneity of variance. Two-tailed *t*-test, χ^2^ test and one-way ANOVA were performed to determine statistically significant differences. *p* < 0.05 or *p* < 0.001 was considered to indicate statistically significant differences (indicated with a single asterisk or double asterisks respectively). Smooth curves were also applied to examine the association between prolactin levels and the prevalence of breast disease subgroups, and to determine whether there was a non-linear relationship. Data were analyzed with the statistical packages R (The R Foundation; http://www.r-project.org; version 3.4.3) and Empower (R) (www.empowerstats.com, X&Y solutions, inc. Boston, Massachusetts).

## Results

### Proportion of hyperprolactinaemia and PRL levels in female patients with breast lesions

**FIGURE 1:**
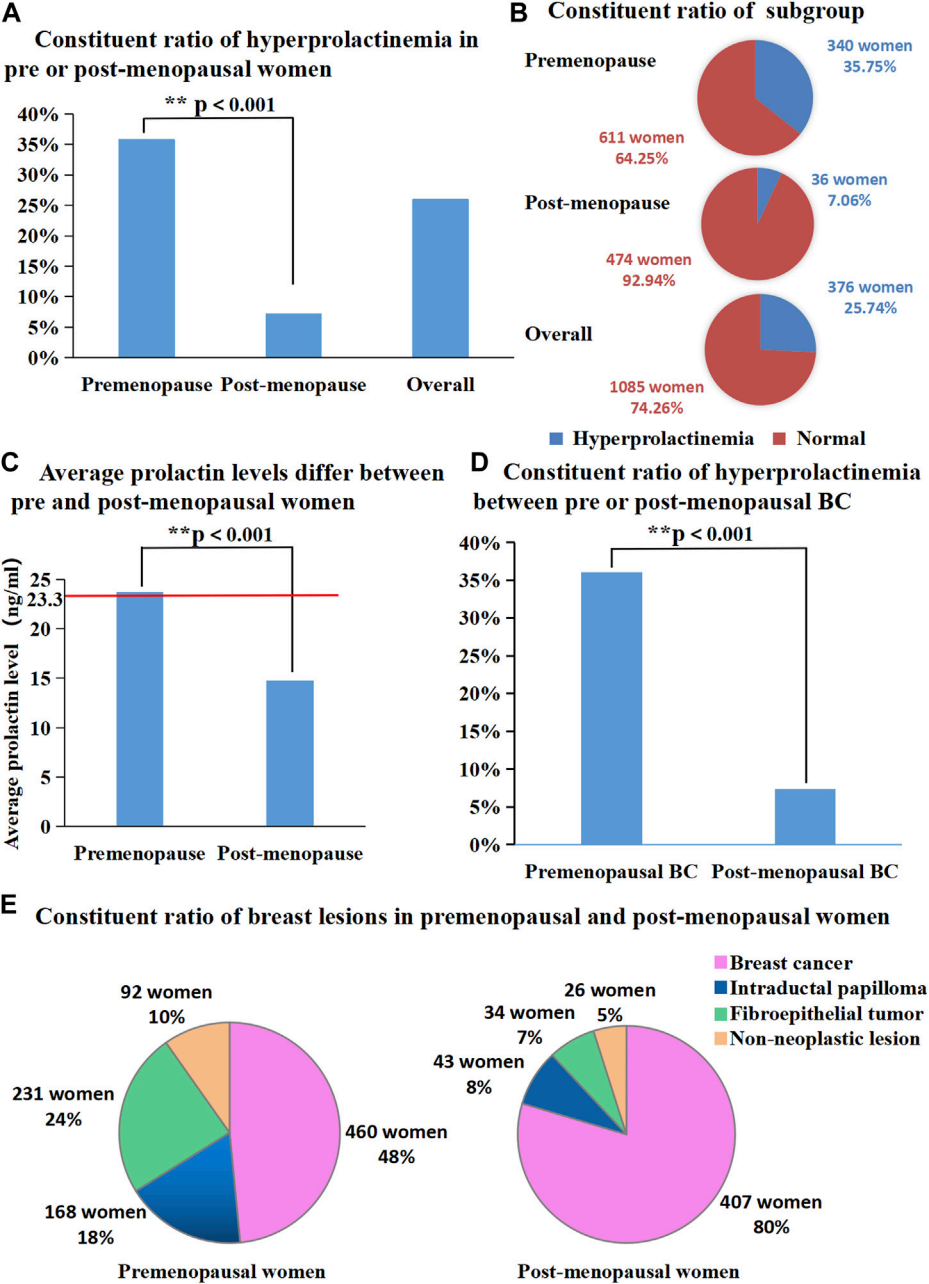
**(A)** Proportion of hyperprolactinemia was significantly different between pre and post-menopausal women (35.75% vs. 7.06%, *p* < 0.001); **(B)** Proportion of each subgroup (number of people with or without hyperprolactinemia); **(C)** Average prolactin levels were significantly different between pre and post-menopausal women (23.72 ng/mL vs. 14.79 ng/mL, *p* < 0.001); **(D)** Proportion of hyperprolactinemia between pre or post-menopausal breast cancer patients. **(E)** Proportion of breast diseases in premenopausal and post-menopausal women.

PRL levels were elevated in 376 of the 1,461 female patients with pathologically diagnosed breast lesions, which accounted for 25.74% of the total number of study patients. The proportion of patients with hyperprolactinaemia showed significant difference between premenopausal (340/951) and postmenopausal (36/510) women (35.75% vs. 7.06%, *p* < 0.001; Figures 1A, B). Additionally, mean serum PRL levels showed significant difference between pre and postmenopausal women (23.72 ng/mL vs. 14.79 ng/mL, *p* < 0.001, Figure 1C). The characteristic features of 1,461 patients are summarized in Tables 1, 2. The proportions of malignant and benign lesions in premenopausal women were almost equal (48% vs. 52%, Figure 1E). However, malignant lesions were dominant in postmenopausal patients as compare to premenopausal patients (80% vs. 20%, Figure 1E). Further analyses revealed that the proportion of hyperprolactinaemia in premenopausal BC patients was significantly higher than that of postmenopausal BC patients (36.09% vs. 7.37%, *p* < 0.001; Figure 1D).

**TABLE 1:** Characteristics of premenopausal women with breast diseases.

Characteristic	*n*	Percent (%)
Overall	951	100
Different age group		
< 35 years old	197	20.72
≥ 35 years old	754	79.28
Different breast diseases in all premenopausal women		
BC	460	48.37
IDP	168	17.67
FET	231	24.29
NNL	92	9.67
Different breast diseases in premenopausal women aged < 35 years old		
BC	54	27.41
IDP	31	15.74
FET	88	44.67
NNL	24	12.18
Different breast diseases in premenopausal women aged ≥ 35 years old		
BC	406	53.85
IDP	137	18.17
FET	143	18.97
NNL	68	9.02
Malignant and benign lesion		
Malignant lesions	463	48.69
Benign lesions	488	51.31
BMI		
< 24 kg/m^2^	496	52.16
≥ 24 kg/m^2^	455	47.84
Unilateral or bilateral disease		
Unilateral	778	81.81
Bilateral	173	18.19
With or without nipple discharge		
With nipple discharge	230	24.19
Without nipple discharge	721	75.81

**TABLE 2:** Characteristics of post-menopausal women with breast diseases.

Characteristic	n	Percent (%)
**Overall**	510	100
**Different breast diseases**		
BC	407	79.80
IDP	43	8.43
FET	34	6.67
NNL	26	5.10
**Malignant and benign lesion**		
Malignant Lesions	407	79.80
Benign Lesions	103	20.20
**BMI**		
< 24 kg/m^2^	266	52.16
≥ 24 kg/m^2^	244	47.84
**Unilateral or bilateral disease**		
Unilateral	459	90.00
Bilateral	51	10.00
**With or without nipple discharge**		
With nipple discharge	63	12.35
Without nipple discharge	447	87.65

### Proportion of patients with hyperprolactinaemia and PRL levels among premenopausal patients with different breast lesions

**FIGURE 2:**
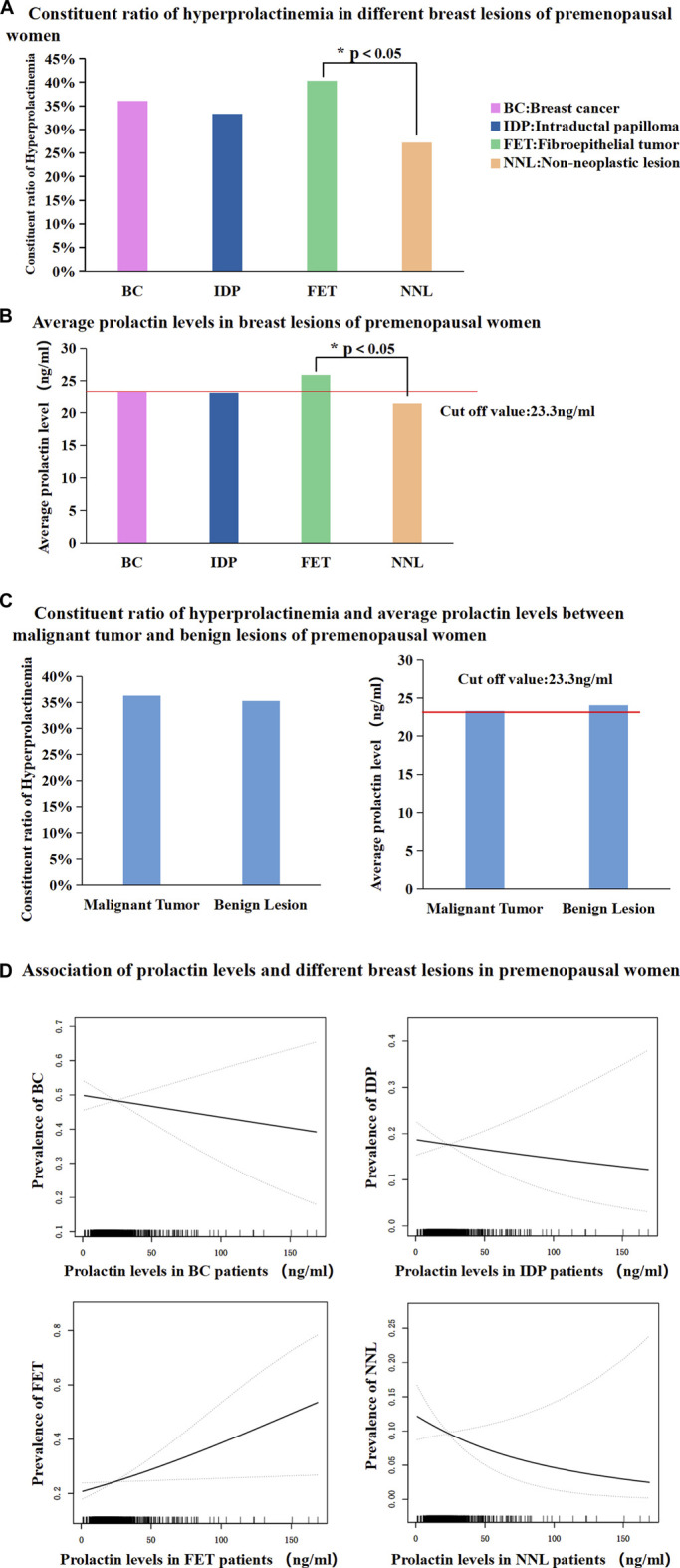
(Continued).

The proportion of patients with hyperprolactinaemia showed no significant difference among BC patients, IDP patients, and non-neoplastic lesion (NNL) patients. The NNL included breast cyst, usual ductal hyperplasia, sclerosing adenosis, lipoma, and ductal ectasia. However, the proportion of patient with FETs was significantly higher than that of patients with NNL (40.24% vs. 27.17%, *p* < 0.05, Figure 2A). Subsequently, mean serum PRL levels in the BC, IDP, and NNL groups showed no significant difference, whereas the mean serum PRL levels were significantly different between FET and NNL patients (25.93 ng/mL vs. 21.42 ng/mL, *p* < 0.05, Figure 2B). The proportion of patients with hyperprolactinaemia (36.29% vs. 35.25%, *p* > 0.05, Figure 2C) and mean serum PRL levels (23.34 ng/mL vs. 24.08 ng/mL, *p* > 0.05 Figure 2C) were not significantly different between malignant and benign lesions in premenopausal women. Furthermore, prolactin level exhibited steady ascending tendency for positive correlation with FET. And the prolactin level exhibited steady descending tendency for negative correlation with NNL. However, there were no apparent correlation between prolactin level and BC, IDP in this study (Figure 2D).

### Proportion of patients with hyperprolactinaemia and PRL levels in pre-menopausal patients of different age groups

**FIGURE 3:**
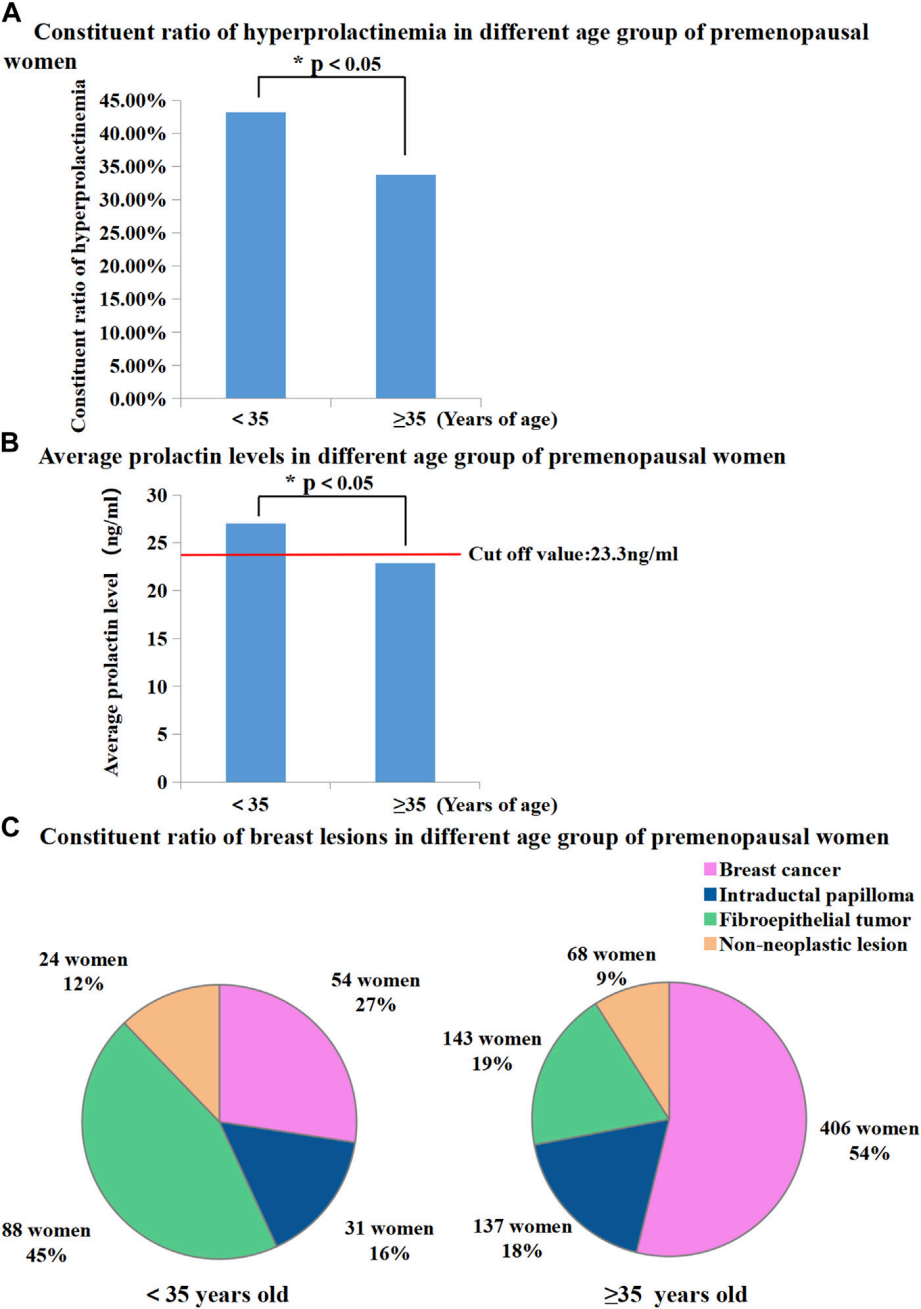
**(A)** Proportion of hyperprolactinemia was significantly different between premenopausal women younger than 35 and older than 35 (43.15% vs. 33.82%, *p* < 0.05); **(B)** Average prolactin levels were significantly different between premenopausal women younger than 35 and older than 35 (27.03 ng/mL vs. 22.86 ng/mL, *p* < 0.01); **(C)** Proportion of breast diseases in different age group of premenopausal women.

BC patients who were younger than 35 years old were defined as “young BC patients”. In this study, 951 premenopausal patients were divided into two groups based on whether in the patients were older or younger than 35 years. The proportion of patients with hyperprolactinaemia showed significant differences between females aged < 35 years and those aged ≥ 35 years (43.15% vs. 33.82%, *p* < 0.05, Figure 3A). Mean serum PRL levels in patients aged < 35 years were higher than those in patients aged ≥ 35 years (27.03 ng/mL vs. 22.86 ng/mL, *p* < 0.05, Figure 3B). In the age group of < 35 years, the dominant breast lesion was FET (45%, Figure 3C), and BC accounted only for approximately a quarter of all patients (27%, Figure 3C). By contrast, BC was the most frequent lesion (54%, Figure 3C) in the age group of ≥ 35 years, and the proportion of patients with FETs decreased dramatically to 19% (Figure 3C). Interestingly, the proportion of patients with IDP in these two age groups was almost similar (16% vs. 18%, Figure 3C).

### Proportion of patients with hyperprolactinaemia and PRL levels in premenopausal patients with different breast lesions in subgroups

**FIGURE 4:**
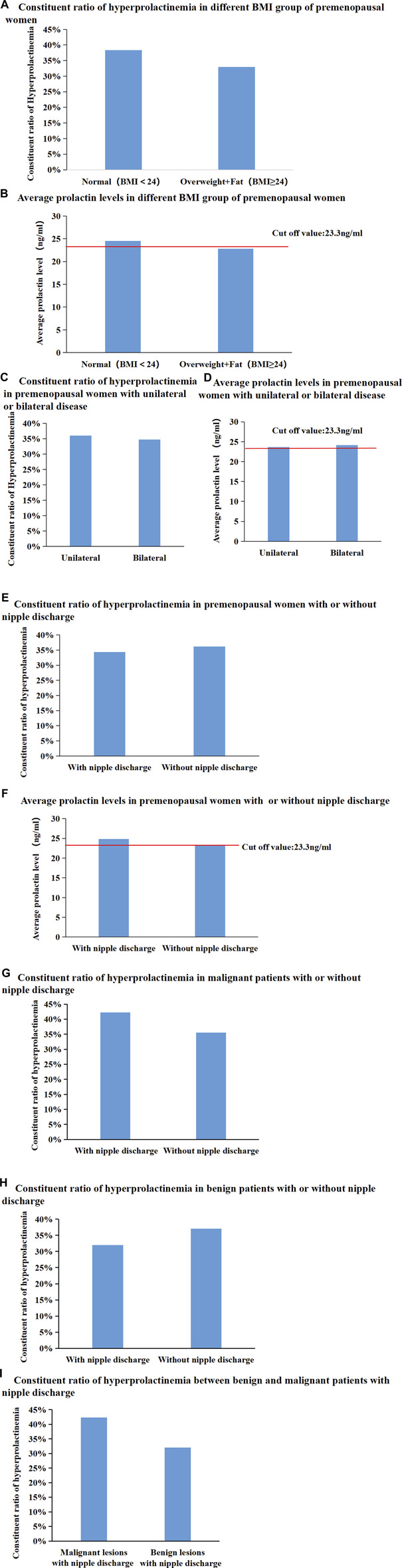
(Continued).

Patients were divided into two groups according to whether their body mass index (BMI) was < 24 or ≥ 24 kg/m^2^. The proportion of patients with hyperprolactinaemia and mean serum PRL levels were not significantly different between the different BMI subgroups (Figures 4A, B). Moreover, the proportion of patients with hyperprolactinaemia and mean serum PRL levels were not significantly different between patients with unilateral breast diseases and those with bilateral breast diseases (Figures 4C, D). In addition, the proportion of patients with hyperprolactinaemia and mean serum PRL levels did not show any significant difference in premenopausal women with or without nipple discharge (Figures 4E, F). Subsequently, in 488 premenopausal patients with malignant breast diseases, the proportion of patients with hyperprolactinaemia in the group with nipple discharge was higher than that in the group without nipple discharge (42.31% vs. 35.52%), but there was no significant difference (*p* = 0.338, Figure 4G). Subsequently, 463 premenopausal patients with benign breast diseases were further divided into two groups based on the presence or absence of nipple discharge and found that there was not significant difference between the groups (32.02% vs. 37.1%, *p* = 0.259, Figure 4H). Among 230 premenopausal patients with nipple discharge, the proportion of patients with hyperprolactinaemia was higher in the malignant disease group than in the benign disease group (42.31% vs. 32.02%), but there was no significant difference between the proportions (*p* = 0.169, Figure 4I). All consitituent ratios of hyperprolactineamia in premenopausal women of different subgroup were summarized in Table 3.

**TABLE 3:** Consitituent ratio of hyperprolactinemia in premenopausal women.

Characteristic	Hyperprolactinemia (%)	Normal (%)	Total
**Pre and post-menopausal women**			
Overall	376 (25.74)	1,085 (74.26)	1,461
Premenopausal	340 (35.75)	611 (64.25)	951
Post-menopausal	36 (7.06)	474 (92.94)	510
**Pre and post-menopausal women with BC**			
Premenopausal BC	166 (36.09)	294 (63.91)	460
Post-menopausal BC	30 (7.37)	377 (92.63)	407
**Different breast diseases in premenopausal women**			
BC	166 (36.09)	294 (63.91)	460
IDP	56 (33.33)	112 (66.67)	168
FET	93 (40.26)	138 (59.74)	231
NNL	25 (27.17)	67 (72.83)	92
**Malignant and benign lesion in premenopausal women**			
Malignant lesions	168 (36.29)	295 (63.71)	463
Benign lesions	172 (35.25)	316 (64.75)	488
**Different age group in premenopausal women**			
< 35 years old	85 (43.15)	112 (56.85)	197
≥ 35 years old	255 (33.82)	499 (66.18)	754
**BMI in premenopausal women**			
< 24 kg/m^2^	190 (38.31)	306 (61.69)	496
≥ 24 kg/m^2^	150 (32.97)	305 (67.03)	455
**Unilateral or bilateral disease in premenopausal women**			
Unilateral	280 (35.99)	498 (64.01)	778
Bilateral	60 (34.68)	113 (65.32)	173
**With or without nipple discharge in premenopausal women**			
With nipple discharge	79 (34.35)	151 (65.65)	230
Without nipple discharge	261 (36.20)	460 (63.80)	721
**With or without nipple discharge in premenopausal women with malignant lesions**			
With nipple discharge	22 (42.31)	30 (57.69)	52
Without nipple discharge	146 (35.52)	265 (64.48)	411
**With or without nipple discharge in premenopausal women with benign lesions**			
With nipple discharge	57 (32.02)	121 (67.98)	178
Without nipple discharge	115 (37.10)	195 (62,90)	310
**Malignant or benign Lesions with nipple discharge in premenopausal women**			
Malignant Lesions	22 (42.31)	30 (57.69)	52
Benign Lesions	57 (32.02)	121 (67.98)	178

Abbreviations: BC, breast cancer; IDP, intraductal papilloma; FET, fibroepithelial tumour; NNL, non-neoplastic lesion.

## Discussion

Our study showed that the proportion of patients with hyperprolactinaemia among a total of 1,461 female breast disease patients was as high as 25.74%. Furthermore, the proportion of patient with hyperprolactinaemia among premenopausal patients with breast disease (35.75%, 340/951) was significantly higher than among postmenopausal patients with breast disease (7.06%, 36/510). Hyperprolactinemia prevalence in the healthy premenopausal population is 4.1%, and the overall prevalence of hyperprolactinaemia in the cohort of searchable study is between 0.23% and 4.1% (Alpanes et al., 2013; Soto-Pedre et al., 2017), close to 7.06% of postmenopausal patients, but dramatically lower than 35.75% of premenopausal patients in our study. Therefore, the phenomenon of a high prevalence of hyperprolactinaemia in Chinese premenopausal patients with breast disease is noteworthy. From our perspective, the possible reasons for the above phenomenon are as follows: First, it was reported that the relative risk of hyperprolactinemia were 2.64 times greater among women who has used oral contraceptives for more than 1 year and 6.25 times greater if this use started before the age of 25 (Badawy et al., 1981). Premenopausal women are more sexually active and therefore may have a higher incidence of hyperprolactinemia due to contraceptive use. Secondly, epidemiological surveys showed that the incidence of prolactinoma in female population started to rise rapidly from the age of 10, peaked around the age of 34, and gradually declined after the age of 35 (Oh et al., 2021). These findings could also explain why the incidence of hyperprolactinemia in premenopausal women is significantly higher than that in postmenopausal women with breast diseases.

Since the 1990s, there has been abundant evidence form *in vitro* and animal studies that PRL plays an important role in the origin and development of BC (Muhlbock and Boot, 1959; Welsch and Nagasawa, 1977). The specific mechanism may be that the combination of PRL and long-form PRLR activates the JAK-STAT related signalling pathway, promotes the mitosis of breast cells, inhibits apoptosis, and induces the angiogenesis of BC cell lines (Sa-Nguanraksa et al., 2021; Yuyi Tang et al., 2022). In addition, data from prospective case control studies indicate that elevated circulating PRL levels are associated with an increased risk of hormone receptor positive BC in postmenopausal women (Tworoger et al., 2013). However, most population-based cohort studies have shown no significant association between hyperprolactinaemia and BC. Our data showed that the proportion of patients with hyperprolactinaemia and mean serum PRL levels were not significantly different between malignant and benign lesions in premenopausal patients. Moreover, there was no significant difference in patient proportion and composition of hyperprolactinaemia among patients with premenopausal BC, IDP and fibrous epithelial tumour. Therefore, our data do not support the association between hyperprolactinaemia and BC.

In the present study, the proportion of patients with hyperprolactinaemia in the FET group was significantly higher than that in the NNL group. Smooth curve analysis indicated that prolactin level was associated with an increased probability of having FET. These results suggested that hyperprolactinaemia may have a certain relationship with the occurrence of breast FETs in Chinese premenopausal women. Breast fibroadenomas and phyllodes tumours are both fibrous epithelial neoplasms, which are a group of heterogeneous bidirectional neoplasms with a proliferation of the epithelial and mesenchymal components. Fibroadenoma is thought to originate from the hormone-dependent stroma of lobules and to be associated with the abnormal response of lobules to oestrogen stimulation. Hyperprolactinaemia can be induced by hyperoestrogenaemia. Mutated PRLRs can be detected in fibroadenomas, and the mutation in the PRLR is associated with increased serum PRL levels (Courtillot et al., 2010). These results suggest that hyperprolactinaemia is associated with the occurrence of FETs. Fibroadenoma is more likely to occur in younger women than in older women, and our data showed that the proportion of patients with hyperprolactinaemia among those with breast diseases who aged < 35 years old was significantly higher than that in patients aged ≥ 35 years old. Furthermore, FETs were observed in nearly half (45%) of the patients with breast diseases younger than 35 years old and only in approximately one-fifth (19%) of patients who were aged ≥ 35 years old. From another viewpoint, it is suggested that hyperprolactinaemia may have relationship with FETs to some extent.

The main physiological function of PRL is to promote the development and growth of mammary gland secretion tissue, initiate and maintain lactation, and increase protein synthesis in mammary glands (Freeman et al., 2000). Breast milk is considered as physiological nipple discharge and secondary galactorrhea is mostly related to elevating circulating PRL levels caused by various reasons, which can be regarded as functional nipple discharge. Pathological nipple discharge is usually serous, bloody, or clear water-like, and the aetiology is usually an intraductal papillary tumour or intraductal carcinoma (Sakorafas, 2001). Our data showed that there was no difference in the proportion of patients with hyperprolactinaemia among premenopausal patients with and without nipple discharge. There was no significant difference in the proportion and composition of patients with hyperprolactinaemia between benign and malignant lesions with nipple discharge. There was no significant difference in the proportion and composition of patients with hyperprolactinaemia between the papilloma group and the NNL group. Therefore, our study does not support a direct association between hyperprolactinaemia and the occurrence of nipple discharge lesions.

The strength of our study is to investigate the proportion of hyperprolactinaemia in a large sample of Chinese patients with breast diseases. While the limitation is that it was a single-center retrospective study. More multi-center and population-based cohort studies should be carried out and might confirm our findings. Another disadvantage is we failed to understand the etiology of hyperprolactinemia. Considering the main issue of our article was not to discuss the relationship between thyroid disease, adenomas of the pituitary or mental drugs and breast diseases, future endocrinology related studies may reveal the answers.

In addition, the reference value range of serum PRL concentration is 4.79 ng/mL (102 μ IU/mL) to 23.30 ng/mL (496 μ IU/mL), this data comes from the reference value standard based on the upper limit of serum PRL level measured by Roche before 2012 for 300 healthy people, including men and non-pregnant women. Therefore, we analyze that, considering the applicability of reference values to their respective patient populations that should be studied by each laboratory, as well as the changes in Chinese people’s eating habits, behaviors, economy and environment over time and with the progress of the times, there may be an overall increase in serum PRL levels. At the same time, it may be that the normal serum PRL levels in Chinese and foreign healthy people are different. Therefore, if necessary, we should establish a reference range that is more suitable for current Chinese women, and we can also independently establish a reference range of serum PRL concentration in different periods of women according to the classification of premenopausal and postmenopausal women, so that we can further study.

In conclusion, our study illustrated a very important phenomenon: a high proportion of patients with hyperprolactinaemia among Chinese female patients with breast diseases, especially in premenopausal women younger than 35 years of age; the occurrence of hyperprolactinaemia in terms of composition and proportion is higher. The proportion of patients with hyperprolactinaemia in breast neoplasms, especially FETs, was significantly higher than that in NNLs. However, this finding does not directly confirm that PRL plays an important role in the occurrence, development, and clinical process of various breast neoplastic diseases, warranting confirmation through further clinical and basic studies. Moreover, if in fact there was a causal relationship, treatment of hyperprolactinaemia may have the potential to reduce the overall incidence of breast disease in premenopausal women and thus reduce healthcare resource consumption.

## Data Availability

The raw data supporting the conclusion of this article will be made available by the authors, without undue reservation.
